# Modified McLaughlin procedure combined with proximal humeral locking plate for proximal humeral fractures with posterior shoulder dislocation and reverse Hill-Sachs lesion: a retrospective case series

**DOI:** 10.3389/fsurg.2026.1881342

**Published:** 2026-08-24

**Authors:** Yi-Fei Wang, Hai-Jian Lu, Xiang Yu, Feng Zhou, Bing-Li Liu, Rong-Guang Ao, Bao-Qing Yu

**Affiliations:** Department of Orthopedics, The Seventh People's Hospital Affiliated to Shanghai University of TCM, Shanghai, China

**Keywords:** locking plate, modified McLaughlin procedure, posterior shoulder dislocation, proximal humeral fracture, reverse Hill-Sachs lesion

## Abstract

**Objective:**

Proximal humeral fractures with posterior shoulder dislocation are rare, high-energy injuries that pose significant treatment challenges. This study aimed to evaluate the clinical application and short-term outcomes of a combined surgical technique utilizing the modified McLaughlin procedure with a proximal humeral locking plate for such complex injuries.

**Methods:**

A retrospective case series was conducted on 9 patients with proximal humeral fractures and posterior shoulder dislocation treated at our hospital from March 2019 to September 2024. All patients underwent combined surgical treatment: structural bone grafting using a pedicled lesser tuberosity fragment, fixed with independent cannulated screw under compression to reconstruct the reverse Hill-Sachs defect, along with fixation of the humeral fracture using a proximal humeral locking plate. Perioperative parameters were recorded, and Visual Analog Scale (VAS), University of California, Los Angeles (UCLA) Shoulder Rating Scale, Constant-Murley scores, shoulder range of motion (ROM), and Limb Symmetry Index(LSI) were assessed at the 1-year postoperative follow-up.

**Results:**

All 9 patients completed a mean follow-up of 15.1 months. The median VAS score was 0, the median UCLA score was 32, and the median Constant-Murley score was 86. Shoulder ROM recovery was satisfactory, with median active forward flexion, abduction, external rotation, and internal rotation measuring 155°, 145°, 60°, and 65°, respectively. No cases of redislocation, implant failure, or avascular necrosis of the humeral head occurred.

**Conclusion:**

For patients with proximal humeral fractures and posterior shoulder dislocation involving a reverse Hill-Sachs defect occupying 25%–40% of the articular surface, treatment with the combined surgical technique of modified McLaughlin procedure combined with locking plate fixation effectively restores shoulder anatomy, results in minimal postoperative pain, and achieves satisfactory functional recovery. This constitutes a reliable treatment option for such complex injuries.

**Level of evidence:**

IV (Retrospective Case Series).

## Introduction

Proximal humeral fractures with posterior shoulder dislocation represent rare yet highly destructive high-energy injuries. Posterior dislocations comprise only 2%–5% of all shoulder dislocations (1, 2), the occurrence of an associated proximal humeral fracture is even less common, presenting significant therapeutic challenge. These injuries are frequently accompanied by a reverse Hill-Sachs lesion—an anteromedial impaction defect of the humeral head. When this defect occupies 25%–50% of the articular surface, maintaining biomechanical stability with simple reduction and internal fixation alone is challenging. Inadequate management carries a high risk of postoperative redislocation and traumatic arthritis (3). This study employed a combined approach utilizing the modified McLaughlin procedure and proximal humeral locking plate fixation. The modified McLaughlin technique was used for structural bone grafting to repair the bony defect and restore joint stability, while the locking plate provided reliable fixation for the proximal humeral fracture. By retrospectively analyzing postoperative pain, shoulder range of motion, functional scores, and complication rates, we systematically evaluated the clinical efficacy of this combined protocol, aiming to provide a more rational strategy and evidence base for managing such complex shoulder trauma.

## Materials and methods

### Study design

This was a retrospective case series. We reviewed the medical records of all consecutive patients presenting with proximal humeral fractures and posterior shoulder dislocation who were treated with the modified McLaughlin procedure combined with a proximal humeral locking plate at our hospital between March 2019 and September 2024. All patients provided informed consent. All procedures complied with the ethical principles of clinical research outlined in the Declaration of Helsinki and were approved by the Medical Ethics Committee of our hospital.

### Inclusion and exclusion criteria

**Figure 1:**
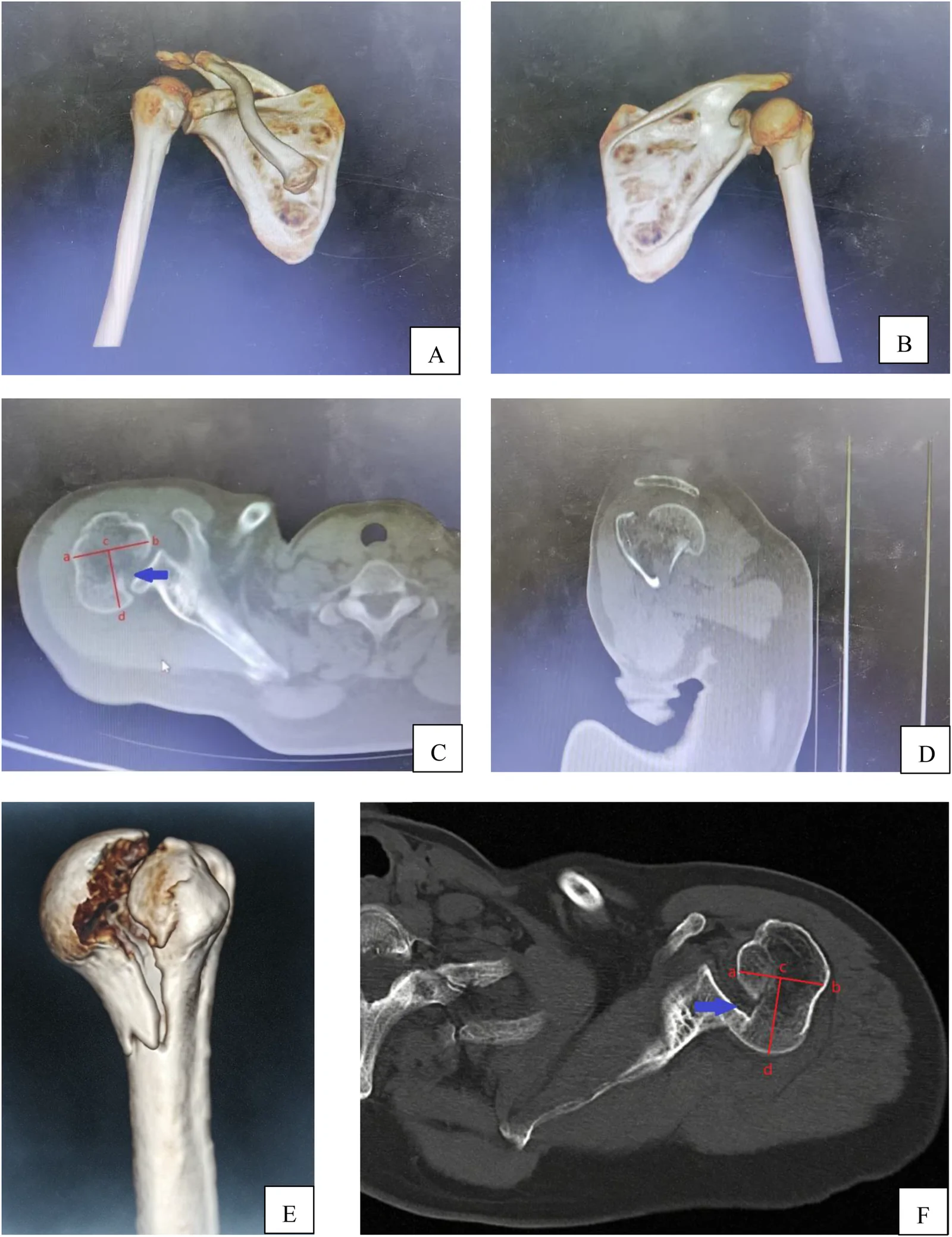
Preoperative 3D CT of the patient NO.4: **(A)** Anterior view. **(B)** Posterior view. **(C)** Axial view. Line ab is the articular surface connecting line, and line cd bisects the articular surface. The blue arrow indicates the reverse Hill-Sachs lesion. The defect area is between 25% and 50%. **(D)** Sagittal view. Preoperative 3D CT of the patient NO.7: **(E)** Anterior view. **(F)** Axial view.

**Inclusion criteria:** 1. Definite history of trauma. 2. Three-dimensional computed tomography (3D CT) confirming proximal humeral fracture with posterior shoulder dislocation. 3. 3D CT demonstrating a reverse Hill-Sachs lesion of the humeral head, with an extent ranging from 25% to 50% of the articular surface (Figure 1). 4. Treatment with open reduction and internal fixation using the modified McLaughlin procedure combined with a proximal humeral locking plate. 5. Age between 18 and 85 years.

**Exclusion criteria:** 1. Pathological fracture. 2. Old fracture. 3. Open fracture. 4. Polytrauma patients. 5. Concomitant major vascular or nerve injury. 6. Incomplete follow-up data.

### Treatment

#### Surgical procedure

Patients were placed in the beach chair position under general anesthesia, with routine sterile preparation and draping. A deltopectoral approach was utilized, preserving the cephalic vein to expose the anterior aspect of the shoulder joint and the proximal humerus fracture site. The long head of the biceps tendon (LHBT) was routinely inspected within the bicipital groove; in the absence of gross pathological changes, the LHBT was preserved without further intervention. The subscapularis tendon was identified at its insertion on the lesser tuberosity; a corticocancellous bone block containing the lesser tuberosity and the attached subscapularis tendon insertion was osteotomized *en bloc* using an oscillating saw, and the intact bone-tendon unit was carefully mobilized and set aside for later use. Fracture hematoma and soft tissue interposed in the fracture site were cleared. Fracture fragments were reduced and temporarily fixed with Kirschner wires. Under C-arm fluoroscopic guidance, satisfactory fracture reduction, alignment, and humeral head position were confirmed. An appropriately sized proximal humeral locking plate was positioned just distal to the tip of greater tuberosity and posterolateral to the bicipital groove. Eight multi-directional locking screws were inserted into the humeral head proximally, and three bicortical screws were placed distally. The reserved lesser tuberosity bone block was then impacted into the anteromedial bony defect of the humeral head and fixed with a cannulated screw under compression inserted along a postero-medial trajectory to optimize graft impaction and avoid intra-articular penetration. Finally, the mobilized subscapularis tendon was reapproximated to the residual humeral metaphysis and securely repaired with non-absorbable sutures to restore physiological resting tension. Passive range of motion of the shoulder joint was performed to confirm stable fixation without impingement. Fluoroscopy was repeated to verify reduction, plate, and screw positions. The wound was thoroughly irrigated with copious normal saline, hemostasis was achieved, a drainage tube was placed, and the incision was closed layer by layer.

#### Postoperative rehabilitation and follow-up

**Figure 2:**
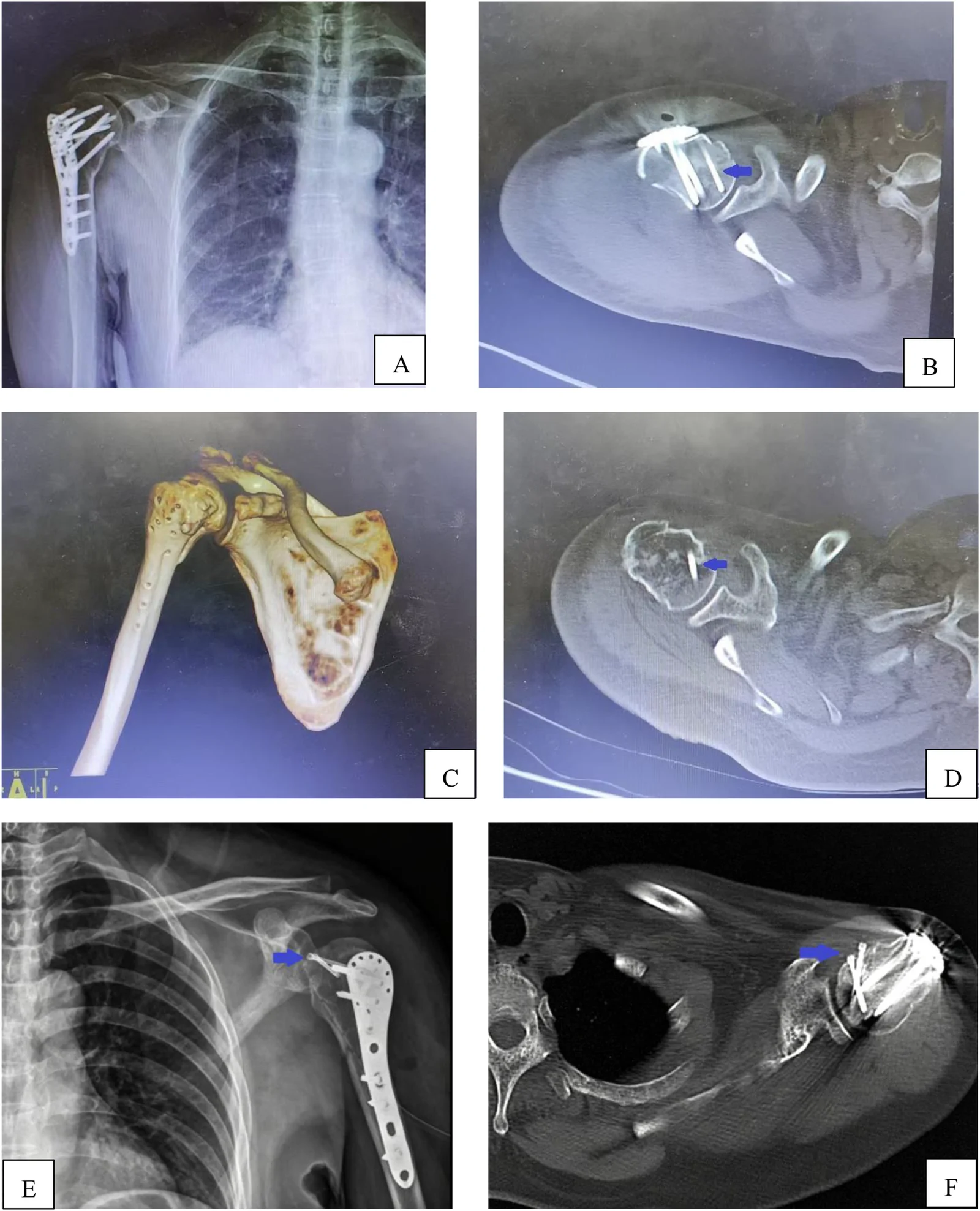
Postoperative imaging of the patient NO.4: **(A)** Anteroposterior x-ray. **(B)** Axial view of 3D CT. The blue arrow indicates the screw used to fix the bone graft. **(C)** 3D CT after removal of internal fixation at 1 year postoperatively. **(D)** The plate was removed, but the screw fixing the bone graft (blue arrow) was retained. Postoperative imaging of the patient NO.7: **(E)** Anteroposterior x-ray. **(F)** Axial view of 3D CT.

A phased postoperative rehabilitation protocol was implemented: 0–6 weeks (Protection Phase): Patients wore an abduction brace. Active hand, wrist, and elbow exercises, along with shoulder pendulum exercises, were encouraged. During this phase, the abduction brace immobilized the shoulder in a position that restricted excessive external rotation, and active external rotation was strictly prohibited to protect the healing lesser tuberosity osteotomy and subscapularis repair. 6–12 weeks (Recovery Phase): The brace was discontinued. Focus shifted to gradually restoring shoulder range of motion through wall-climbing exercises and assisted training, initiating isometric strengthening of the rotator cuff muscles. > 12 weeks (Strengthening Phase): Resistance training with elastic bands and dumbbells, along with functional activities like dressing and swimming, were permitted to comprehensively restore muscle strength, endurance, and joint stability. The entire rehabilitation process adhered to the principle of “pain-free progression.” Clinical and radiographic follow-up evaluations were scheduled at 1, 3, 6, and 12 months postoperatively to assess fracture healing. Hardware removal was typically considered at approximately 12 months post-surgery (Figure 2).

### Observation indicators

#### Baseline indicators

Collected demographic and clinical data included: gender, age, side of fracture, cause of injury, the Neer classification of the proximal humeral fracture, the time interval from injury to surgery, and the size of the reverse Hill-Sachs defect.

The size of the reverse Hill-Sachs defect was measured according to the method described by Cicak et al. (4). On axial CT images, the slice demonstrating the maximal impression of the humeral head was selected. A line was drawn connecting the anterior and posterior articular margins of the humeral head. This line was then divided into four equal segments, with each segment representing 25% of the total articular surface area. The percentage of the defect was estimated by referencing these quadrants. Two independent senior surgeons performed the measurements separately, and consensus was reached for each case (Figure 1).

#### Outcome indicators

Shoulder range of motion (ROM), Fracture Union Time, Visual Analog Scale (VAS) score, University of California, Los Angeles (UCLA) Shoulder Rating Scale score, Constant-Murley score, and Limb Symmetry Index (LSI) were recorded at the 1-year postoperative follow-up.

Postoperative complications were systematically evaluated at each follow-up visit, including surgical site infection, implant loosening or failure, redislocation, avascular necrosis of the humeral head, and traumatic arthritis.

Shoulder ROM was measured using a standard goniometer with the patient seated or standing. Evaluations included: active forward flexion, active abduction, active internal rotation (assessed as the highest spinal spinous process reachable by the thumb with the hand behind the back, converted to degrees via the Constant-Murley scale), and active external rotation (measured in seated patients with the shoulder abducted 90°, elbow flexed 90°, and forearm vertical. A goniometer axis was aligned at the olecranon, with the stationary arm perpendicular to the floor and moving arm parallel to the ulna). All measurements started from the neutral position (0 degrees), recording the maximum angle of active movement.

Fracture union was confirmed on follow-up 3D CT, defined as complete obliteration of the fracture line, continuous trabecular bone bridging the fracture site, absence of implant loosening or failure, and negative longitudinal percussion tenderness of the affected limb. Two independent senior radiologists performed image interpretation separately; any discrepancies were resolved via joint review. Time to union was calculated as the interval from the date of surgery to the date of CT-confirmed union.

The UCLA score integrates subjective and objective assessments, totaling 35 points across five domains: pain (10 points), function (10 points), active forward flexion (5 points), forward flexion strength (5 points), and patient satisfaction (5 points). Overall efficacy was graded as: Excellent (34–35 points), Good (28–33 points), Fair (21–27 points), and Poor (≤ 20 points).

LSI was calculated as: (Operated side ROM)/(Contralateral healthy side ROM) × 100%. LSI values were interpreted as: ≥90% = excellent symmetry, 85%–89% = good symmetry, 70%–84% = fair symmetry, <70% = poor symmetry.

#### Statistical analysis

All data were managed and analyzed using SPSS software (version 26.0). Continuous variables are presented as median (interquartile range, IQR) or mean ± standard deviation, as appropriate. Categorical variables are reported as number (percentage). Inferential statistics and 95% confidence intervals were omitted to avoid overinterpretation. Formal sample size calculation was not performed, as this study aimed to describe surgical outcomes for a rare injury rather than to test a prespecified hypothesis.

## Results

### Baseline characteristics

Nine eligible patients with proximal humeral fractures and posterior shoulder dislocation were enrolled. There were 5 males and 4 females, with a median age of 45 years (range: 28–68 years). Regarding injury mechanisms, most patients sustained injuries from falls, while others resulted from high-energy trauma such as traffic accidents. Laterality was nearly equal, with 5 left-sided and 4 right-sided fractures. The average interval from injury to surgery was short (mean 1.4 ± 0.5 days), with 55.6% of patients undergoing surgery within 24 h of injury. All patients presented with a typical reverse Hill-Sachs lesion of the humeral head, with a defect size ranging from 25% to 40% (mean 31.1% ± 6.0%) (Table 1).

**Table 1:** Clinical data comparison.

No.	Gender	Age (y)	Fracture side	The cause of injury	NEER classification	Time from injury to surgery (d)	Reverse Hill-Sachs defect (%)
1	Male	60	Left	Fall	II-part(Surgical neck)	2	25
2	Female	68	Right	Fall	III-part(Surgical neck + Lesser tuberosity)	2	30
3	Male	36	Left	Traffic accident	II-part(Surgical neck)	1	25
4	Male	45	Right	Traffic accident	III-part(Surgical neck)	1	40
5	Female	63	Left	Fall	II-part(Surgical neck + Greater tuberosity)	1	40
6	Male	58	Right	Fall	II-part(Surgical neck)	1	30
7	Female	59	Left	Fall	II-part(Surgical neck)	2	35
8	Female	45	Right	Traffic accident	II-part(Surgical neck)	1	30
9	Male	28	Right	Traffic accident	III-part(Surgical neck + Greater tuberosity)	2	25

### Outcome measurements

All patients completed follow-up, with a mean follow-up duration of 15.1 ± 1.8 months. All 9 patients achieved solid bony union, with a median time to union of 12.22 ± 1.39 weeks. No postoperative complications occurred during the entire follow-up period, including surgical site infection, implant loosening/failure, redislocation, avascular necrosis of the humeral head, or traumatic arthritis. All patients underwent uneventful removal of the proximal humeral locking plate at 12 months postoperatively. No implant-related symptoms were observed throughout the entire follow-up period.

At the 1-year postoperative follow-up, patients demonstrated good recovery of shoulder function. Regarding ROM of the operated shoulder, the median values were 155°(IQR: 145°–162.5°) for forward flexion, 145°(IQR: 125°–150°) for abduction, 60°(IQR: 50°–65°) for external rotation, and 65°(IQR: 60°–75°) for internal rotation. To account for individual variability, we calculated the LSI relative to the contralateral healthy shoulder. The median LSI was 91% (IQR: 85%–91%) for forward flexion, 85% (IQR: 79%–86%) for abduction, 72% (IQR: 59%–76%) for external rotation, and 69% (IQR: 67%–82%) for internal rotation, indicating favorable restoration of physiological symmetry, particularly for elevation movements. The median VAS score was 0(IQR: 0–1), indicating excellent postoperative pain control. Functional scores showed a median UCLA score of 32(IQR: 30–33), corresponding to a “Good” rating. The median Constant-Murley score was 86(IQR: 77–91.5), reflecting satisfactory restoration of shoulder stability and daily function (Table 2).

**Table 2:** Comparison of outcome indicators.

No.	Follow-up time (m)	Fracture Union Time (wk)	Range of motion(affected side/unaffected side/LSI)				VAS	UCLA	Constant-Murley
			flexion	abduction	external rotation	internal rotation			
1	15	11	160/170/94%	145/165/88%	65/90/72%	65/90/72%	0	33	88
2	14	12	150/180/83%	150/175/86%	70/90/78%	80/90/89%	0	34	92
3	18	14	170/180/94%	150/180/83%	60/80/75%	70/85/82%	0	33	94
4	16	12	155/170/91%	145/170/85%	55/85/65%	50/80/63%	1	31	84
5	18	14	160/175/91%	140/175/80%	50/85/59%	60/90/67%	0	32	85
6	17	13	130/160/81%	125/160/78%	45/85/53%	60/90/67%	1	29	76
7	16	13	145/165/88%	105/165/63%	50/90/56%	55/80/69%	3	30	70
8	13	11	165/180/92%	160/180/89%	65/85/76%	75/85/88%	2	33	92
9	13	10	145/170/85%	135/170/79%	70/90/78%	80/90/89%	0	30	86

VAS, visual analogue scale; UCLA, University of California, Los Angeles shoulder rating scale; LSI, limb symmetry index.

## Discussion

Proximal humeral fractures combined with posterior shoulder dislocation constitute rare, high-energy injuries, representing a diagnostic and therapeutic challenge in orthopedic trauma. While posterior dislocations account for only 2%–5% of all shoulder dislocations, the incidence of concomitant fractures is markedly lower, posing a distinct therapeutic challenge (5). The pathological hallmark involves axial compression of the humeral head, resulting not only in fracture displacement but also in an anteromedial “reverse Hill-Sachs” impaction defect (6). Failure to effectively reconstruct this defect severely compromises shoulder mechanical stability and increases the risk of postoperative redislocation and traumatic arthritis (2). The size of the reverse Hill-Sachs defect is a critical determinant of the surgical strategy; defects involving 25%–50% of the articular surface exceed the scope of conservative management or simple fixation and necessitate bone grafting reconstruction (7, 8).

Surgical strategies for reverse Hill-Sachs lesions have evolved, with the core objective being the optimization of mechanical stability and promotion of osseous healing (9). In 1952, McLaughlin first described the classic tendon transfer technique, involving transposition of the subscapularis tendon into the anteromedial defect to act as a dynamic stabilizing filler. Subsequently, Neer, Hughes, and others modified this technique, advocating transfer of the lesser tuberosity along with the tendon to provide stronger bony support for healing (10, 11).

Most published studies on the modified McLaughlin procedure have focused on isolated posterior shoulder dislocation, with the majority enrolling chronic or neglected cases. Berk et al. systematically reviewed 9 studies comprising 97 shoulders, 8 of which involved chronic locked posterior dislocation (time from dislocation to surgery: 8–25 weeks), reporting a weighted mean Constant-Murley score of 75 and UCLA shoulder score of 29 (12). In contrast, Banerjee et al. reported a mean Constant score of 92 in a pure acute cohort (time to surgery <3 weeks) (13). Our series differs in that all cases were treated acutely (mean 1.4 days post-injury) and concurrently presented with concomitant proximal humeral fractures. We adopted a combined surgical technique combining the modified McLaughlin procedure with a proximal humeral locking plate. Firstly, structural bone grafting using a pedicled lesser tuberosity fragment, fixed with a cannulated screw under compression, specifically addresses the bone defect and restores the spherical contour of the humeral head (14). Secondly, the proximal humeral locking plate, utilizing its angular stability, is dedicated to maintaining the overall structural integrity of the proximal humerus fracture. This combined approach allows for safe, progressive rehabilitation in the early postoperative period. Through a retrospective case series of 9 patients treated with this combined technique, this study further explores its clinical value and efficacy for such complex shoulder injuries.

Our results suggested that the combined modified McLaughlin procedure and locking plate fixation achieved reliable clinical efficacy and satisfactory functional outcomes in patients with proximal humeral fractures and posterior dislocation. Regarding pain control, the VAS score was 0 at the 1-year postoperative follow-up, indicating thorough pain relief. In terms of shoulder function, the UCLA shoulder score reached 32, and the median Constant-Murley score was 86, suggesting favorable recovery of overall shoulder function, muscle strength, and activities of daily living. Recovery of shoulder ROM was also satisfactory, with median values of 155° for active forward flexion, 145° for abduction, 60° for external rotation, and 65° for internal rotation. The LSI values for forward flexion and abduction were 91% and 85%, respectively, suggesting favorable functional recovery relative to the contralateral side. In contrast, the LSI for rotational movements ranged from 69% to 72%, indicating moderate recovery. Collectively, the restoration of these primary shoulder motion arcs largely returned function to near-physiological levels, sufficiently meeting the demands of daily activities such as dressing, eating, grooming, and working.

The good prognosis in these patients stems from precise anatomical reconstruction. When the defect exceeds 20%–25%, the humeral head is prone to “engagement” with the posterior glenoid rim, leading to difficult reduction or postoperative instability (7, 15). The patients in our cohort had defects ranging from 25%–40%, falling precisely within this critical threshold. Compared to simple open reduction and internal fixation (ORIF) that neglects defect filling, or the classic McLaughlin technique which provides non-anatomical filling, our combined strategy utilizes a pedicled lesser tuberosity for structural bone grafting. This not only restores the spherical contour of the humeral head but also offers biological viability conducive to defect healing. Furthermore, the angular stability design of the locking plate effectively resists shear forces induced by rotator cuff traction. The absence of redislocation or fixation failure in our series strongly corroborates the reliability of this technique in interrupting the vicious cycle of “bone defect - instability - impingement.”

However, Case 7 exhibited a notably poorer outcome, with a Constant-Murley score of 70, a VAS score of 3, and limited abduction. We consider that this poorer prognosis could potentially be associated with underlying osteoporosis and suboptimal rehabilitation compliance. Age-related bone loss likely compromised implant purchase, suggesting that for patients with poor bone quality, adjunctive reinforcement measures and stricter rehabilitation protocols may be necessary.

The timing of surgical intervention is closely linked to functional prognosis and serves as an important independent factor influencing clinical efficacy and long-term quality of life (16). In our study, the average time from injury to surgery was only 1.4 days, with 55.6% of patients undergoing emergency reduction and surgery within 24 h, indicating high temporal efficiency. For shoulder dislocation, early anatomical reduction and surgical stabilization rapidly release entrapped soft tissues, significantly reduce pathologically elevated intra-articular pressure, effectively relieve compression and spasm of vessels supplying the humeral head, minimize the duration of ischemia and hypoxia, improve local microcirculation, and mechanistically reduce the risk of severe complications like avascular necrosis of the humeral head and traumatic arthritis (17, 18). Simultaneously, early emergency surgery mitigates secondary traction injuries to surrounding ligaments, tendons, and neurovascular structures caused by the dislocated state, creating favorable conditions for good postoperative recovery of ROM, muscle strength, and daily function.

### Limitations

This study has several limitations. Firstly, due to the extremely low incidence of proximal humeral fractures with posterior shoulder dislocation, only 9 patients were included. The small sample size and single-center retrospective design limit statistical power. Secondly, owing to the rarity of such cases, no concurrent control group was established, precluding direct comparison of efficacy between different surgical techniques. Thirdly, the follow-up period was relatively short (mean 15.1 months). While sufficient for assessing short-term bone healing and functional recovery, it is inadequate for observing long-term complications such as traumatic arthritis or late collapse of the grafted bone. Additionally, the wide age range (28–68 years) introduces individual variability that may confound prognostic assessment.

## Conclusion

This study demonstrates that for patients with proximal humeral fractures and posterior shoulder dislocation involving a reverse Hill-Sachs defect occupying 25%–40% of the humeral head articular surface, treatment with the modified McLaughlin procedure combined with a proximal humeral locking plate effectively reconstructs the anteromedial humeral head defect. Postoperatively, patients experience minimal pain and satisfactory functional recovery, establishing this combined approach as an effective treatment option for such complex injuries.

## Data Availability

The raw data supporting the conclusions of this article will be made available by the authors, without undue reservation.
